# Supplementary material for: Early infant diagnosis testing for HIV in a hard-to-reach fishing community in Uganda
Source: PLoS One. 2023 Jun 7;18(6):e0268416. doi: 10.1371/journal.pone.0268416 (PMC10246781; doi:10.1371/journal.pone.0268416)
Supplement: S1 File — (PDF) [file pone.0268416.s002.pdf]

ADHERENCE TO EID TESTING PROTOCCOLS IN BUVUMA DISTRICT

GENERAL INFORMATION

STUDY TITTLE: ADHERENCE TO EARLY INFANT DIAGNOSIS OF HIV TESTING PROTOCOL AMONG HIV EXPOSED INFANTS IN BUVUMADISTRICT, UGANDA.

Date of data extraction

yyyy-mm-dd ______ hh:mm ______

File number

______

Name of the health facility

- ◯ Buvuma Health Centre IV
- ◯ Busamuzi Health Centre III
- ◯ Namatale Health Centre III
- ◯ Bugaya Health Centre III
- ◯ Lwajje Health Centre II
- ◯ Lubya Health Centre II

Social Demographic Characteristics of the mother

Age of the mother (Indicate 00 in case of missing data)

______

Marital status

- ◯ Married
- ◯ Single
- ◯ Widowed
- ◯ No data available

Mother already on ART

- ◯ Yes
- ◯ No
- ◯ No data available

If yes, date, month and year of enrolment on ART

yyyy-mm-dd

Estimated distance from Health facilities

- ◯ < 5kilometers
- ◯ > 5kilometers
- ◯ No data available

HIV testing and Enrolment onto ART

When was the mother diagnosed with HIV?

- ◯ Before pregnancy
- ◯ During pregnancy
- ◯ During labour
- ◯ During PNC
- ◯ Unknown
- ◯ Other
- ◯ No data available

Specify

Where did the mother deliver?

- ◯ Home
- ◯ Health facility
- ◯ TBA

Name of health facility where mother delivered from

Ownership of health care facility where mother delivered from

- ◯ Public
- ◯ Private

When did the mother enrol for ART?

- ◯ Before pregnancy
- ◯ During pregnancy
- ◯ During labour
- ◯ During PNC
- ◯ Un known
- ◯ Other

Specify

____

What is the dosing of mother's ARVs?

- ◯ Once a day
- ◯ Twice a day
- ◯ Other

Specify

____

Does the mother have a treatment supporter?

- ◯ Yes
- ◯ No

Did the mother disclose her ART status to the treatment supporter?

- ◯ Yes
- ◯ No

Infant demographics

Exposed infant file number

____

Date infant file was opened

yyyy-mm-dd

Year of Enrolment

yyyy-mm-dd

Sex

- ◯ Male
- ◯ Female

Date of Birth/ date of delivery

yyyy-mm-dd

Name of the clinic where infant was refered from

Infant feeding

- ◯ Exclusive breast feeding
- ◯ Replacement feeding
- ◯ Mixed feeding below 6 months
- ◯ Complementary feeding above 6 months
- ◯ Wean from feeding
- ◯ No longer breast feeding
- ◯ Infant formular

Immunisation status of infant

- ◯ BCG
- ◯ DPT 1
- ◯ DPT 2
- ◯ DPT 3
- ◯ Missing data

Date 1st PCR was collected

yyyy-mm-dd

Date 1st PCR was dispatched

yyyy-mm-dd

Date results were received at the health facility

yyyy-mm-dd

Date results given to the care giver

yyyy-mm-dd

Outcome of the 1st PCR

- Positive
- Negative
- Missing data

Date 1st PCR was repeated

yyyy-mm-dd

Date results of repeated 1st PCR were dispatched

yyyy-mm-dd

Was a 2nd PCR collected?

- Yes
- No

Date 2nd PCR collected

yyyy-mm-dd

Date 2nd PCR was dispatched

yyyy-mm-dd

Age at 2nd DBS

Results of 2nd DBS

- Positve
- Negative
- Not available/ missing

Date results are received at the health facility

yyyy-mm-dd

Date results are given to the caregier

yyyy-mm-dd

Outcome of 2nd PCR test

- Positive
- Negative
- No data

Date 2nd PCR was repeated

yyyy-mm-dd

Date rapid diagnostic test was done

yyyy-mm-dd

Age of infant at the time of rapid diagnostic test

Result of rapid diagnostic test

- Positive
- Negative
- No data available

Infant follow up method

- First attempt
- Second attempt
- Third attemp

Date of attempt

yyyy-mm-dd

Final outcome of infant

- Died
- Lost to follow up
- Discharged negative
- Enrolled into care
- Transferred out

Final outcome of infant

- ◯ Died
- ◯ Lost to follow up
- ◯ Discharged negative
- ◯ Enrolled into care
- ◯ Transferred out

If enrolled, is PRE ART number available

- ◯ Yes
- ◯ No

Was infant linked to care?

- ◯ Yes
- ◯ No

END OF THE FORM
