# Supplementary material for: Early infant diagnosis testing for HIV in a hard-to-reach fishing community in Uganda
Source: PLoS One. 2023 Jun 7;18(6):e0268416. doi: 10.1371/journal.pone.0268416 (PMC10246781; doi:10.1371/journal.pone.0268416)
Supplement: Attachment — Submitted filename: PONE-D-22-12468.pdf [file pone.0268416.s004.pdf]

PLOS ONE

Adherence to the HIV early infant diagnosis testing protocol among HIV exposed infants in a hard-to-reach fishing community in Uganda

--Manuscript Draft--

Manuscript Number:	PONE-D-22-12468
Article Type:	Research Article
Full Title:	Adherence to the HIV early infant diagnosis testing protocol among HIV exposed infants in a hard-to-reach fishing community in Uganda
Short Title:	Adherence to the HIV early infant diagnosis testing protocol among HIV exposed infants
Corresponding Author:	Tonny Ssekamatte, MPH<br>Makerere University School of Public Health<br>Kampala, UGANDA
Keywords:	Diagnosis; Infant; Hard-to-reach; HIV; Uganda
Abstract:	Background<br>Infants born to HIV-infected mothers are at a high risk of acquiring the infection. The World Health Organization (WHO) recommends early diagnosis of HIV-exposed infants (HEIs) through deoxyribonucleic acid polymerase chain reaction (DNA PCR) and rapid HIV testing. Early detection of paediatric HIV is critical for access to antiretroviral therapy treatment (ART) and child survival. There's, however, limited evidence of the adherence to early infant diagnosis (EID) of HIV testing protocol among HEIs in fishing communities in Uganda. This study assessed adherence to EID of HIV testing protocol among HIV-exposed infants in a hard-to-reach fishing community in Uganda.<br>Methods<br>We conducted a cross-sectional study employing quantitative data collection methods among HEIs in selected healthcare facilities in Buvuma islands, Buvuma district. We obtained secondary data from mother-infant pair files enrolled on the EID program using a data extraction tool. Data were analysed using STATA Version 14. Modified poisson regression analysis was used to determine the factors associated with non-adherence to the 1 st DNA PCR test among HIV-exposed infants enrolled into care.<br>Results<br>None of the HIV-exposed infants had done all the EID tests prescribed by the HIV testing protocol within the recommended time frame for the period of January 2014-December 2016. Adherence to the 1 st and 2 nd DNA PCR, and rapid HIV tests was 39.5%, 6.1% and 81.0% respectively. Being under the care of single mothers (PR=1.11, 95% CI: 1.01-1.23, p=0.023) and cessation of breast feeding (PR=0.90, 95% CI: 0.83-0.98, p=0.025) were significantly associated with non-adherence to the 1 st DNA PCR.<br>Conclusion<br>None of the HIV-exposed infants adhered to all the EID tests of HIV testing protocol. Adherence to the 1st DNA PCR was positively associated with being a single mother and exclusive breast feeding. Therefore, single mothers and those who stop breastfeeding should be supported to ensure timely EID.
Order of Authors:	Tonny Ssekamatte, MPH
	Remegio Ndyanabo, MPH
	Aisha Nalugya
	Mary Nakafero, Msc
	Angela Kisakye, MPH
	Aggrey David Mukose, PhD
Additional Information:	
Question	Response
Financial Disclosure	The author(s) received no specific funding for this work.

Powered by Editorial Manager® and ProduXion Manager® from Aries Systems Corporation

Enter a financial disclosure statement that describes the sources of funding for the work included in this submission. Review the submission guidelines for detailed requirements. View published research articles from *PLOS ONE* for specific examples.<br><br>This statement is required for submission and **will appear in the published article** if the submission is accepted. Please make sure it is accurate.<br><br>**Unfunded studies**<br>Enter: *The author(s) received no specific funding for this work.*<br><br>**Funded studies**<br>Enter a statement with the following details:<br>• Initials of the authors who received each award<br>• Grant numbers awarded to each author<br>• The full name of each funder<br>• URL of each funder website<br>• Did the sponsors or funders play any role in the study design, data collection and analysis, decision to publish, or preparation of the manuscript?<br>• **NO** - Include this sentence at the end of your statement: *The funders had no role in study design, data collection and analysis, decision to publish, or preparation of the manuscript.*<br>• **YES** - Specify the role(s) played.<br><br>* typeset	
**Competing Interests**<br><br>Use the instructions below to enter a competing interest statement for this submission. On behalf of all authors, disclose any competing interests that could be perceived to bias this work—acknowledging all financial support and any other relevant financial or non-financial competing interests.<br><br>This statement is required for submission and **will appear in the published article** if	he authors have declared that no competing interests exist.

the submission is accepted. Please make sure it is accurate and that any funding sources listed in your Funding Information later in the submission form are also declared in your Financial Disclosure statement.<br><br>View published research articles from *PLOS ONE* for specific examples.<br><br>**NO authors have competing interests**<br><br>Enter: *The authors have declared that no competing interests exist.*<br><br>**Authors with competing interests**<br><br>Enter competing interest details beginning with this statement:<br><br>*I have read the journal's policy and the authors of this manuscript have the following competing interests: [insert competing interests here]*<br><br>* typeset	
**Ethics Statement**<br><br>Enter an ethics statement for this submission. This statement is required if the study involved:<br><br>• Human participants<br>• Human specimens or tissue<br>• Vertebrate animals or cephalopods<br>• Vertebrate embryos or tissues<br>• Field research<br><br>Write "N/A" if the submission does not require an ethics statement.<br><br>General guidance is provided below. Consult the submission guidelines for detailed instructions. **Make sure that all information entered here is included in the Methods section of the manuscript.**	This study was reviewed and approved by the Makerere University School of Public Health Higher Degrees and Research Ethics Committee (MakSPH HDREC). Permission to conduct this study was also sought from Buvuma District Local government.

**Format for specific study types**<br><br>**Human Subject Research (involving human participants and/or tissue)**<br>• Give the name of the institutional review board or ethics committee that approved the study<br>• Include the approval number and/or a statement indicating approval of this research<br>• Indicate the form of consent obtained (written/oral) or the reason that consent was not obtained (e.g. the data were analyzed anonymously)<br><br>**Animal Research (involving vertebrate animals, embryos or tissues)**<br>• Provide the name of the Institutional Animal Care and Use Committee (IACUC) or other relevant ethics board that reviewed the study protocol, and indicate whether they approved this research or granted a formal waiver of ethical approval<br>• Include an approval number if one was obtained<br>• If the study involved *non-human primates*, add *additional details* about animal welfare and steps taken to ameliorate suffering<br>• If anesthesia, euthanasia, or any kind of animal sacrifice is part of the study, include briefly which substances and/or methods were applied<br><br>**Field Research**<br><br>Include the following details if this study involves the collection of plant, animal, or other materials from a natural setting:<br>• Field permit number<br>• Name of the institution or relevant body that granted permission	
**Data Availability**<br><br>Authors are required to make all data underlying the findings described fully available, without restriction, and from the time of publication. PLOS allows rare exceptions to address legal and ethical concerns. See the PLOS Data Policy and FAQ for detailed information.	Yes - all data are fully available without restriction

Powered by Editorial Manager® and ProduXion Manager® from Aries Systems Corporation

A Data Availability Statement describing where the data can be found is required at submission. Your answers to this question constitute the Data Availability Statement and **will be published in the article**, if accepted.<br><br>**Important:** Stating 'data available on request from the author' is not sufficient. If your data are only available upon request, select 'No' for the first question and explain your exceptional situation in the text box.<br><br>Do the authors confirm that all data underlying the findings described in their manuscript are fully available without restriction?	
**Describe where the data may be found in full sentences. If you are copying our sample text, replace any instances of XXX with the appropriate details.**<br><br>• If the data are **held or will be held in a public repository**, include URLs, accession numbers or DOIs. If this information will only be available after acceptance, indicate this by ticking the box below. For example: *All XXX files are available from the XXX database (accession number(s) XXX, XXX.)*.<br>• If the data are all contained **within the manuscript and/or Supporting Information files**, enter the following: *All relevant data are within the manuscript and its Supporting Information files.*<br>• If neither of these applies but you are able to provide **details of access elsewhere**, with or without limitations, please do so. For example:<br><br>*Data cannot be shared publicly because of [XXX]. Data are available from the XXX Institutional Data Access / Ethics Committee (contact via XXX) for researchers who meet the criteria for access to confidential data.*<br><br>*The data underlying the results presented in the study are available from (include the name of the third party*	All relevant data are within the manuscript and its Supporting Information files

and contact information or URL).<br>• This text is appropriate if the data are owned by a third party and authors do not have permission to share the data.<br><br>* typeset	
Additional data availability information:	

Manuscript Click here to access/download;Manuscript;Manuscript-Adherence plos one.docx

Adherence to the HIV early infant diagnosis testing protocol among HIV exposed infants in a hard-to-reach fishing community in Uganda

Ndyanabo Remegio[1,2], Aisha Nalugya[1], Tonny Ssekamatte[1]*, Mary Nakafero[3], Angela Kisakye[4], Aggrey David Mukose[3]

Institutional affiliation

[1]Department of Disease Control and Environmental Health, Makerere University School of Public Health, Kampala, Uganda; [2]Buvuma District Local government, Lugazi, Uganda; [3]Department of Epidemiology and Biostatistics, Makerere University School of Public Health, Kampala, Uganda; [4]Department of Health Policy Planning and Management, Makerere University School of Public Health, Kampala, Uganda, Kampala, Uganda

Correspondence: Tonny Ssekamatte

Postal address: New Mulago Hospital complex, P.O Box 7072 Kampala, Uganda

Mobile Telephone: +256 782 381484

Email: ssekamattet.toca@gmail.com ; tssekamatte@musph.ac.ug

Abstract

Background

Infants born to HIV-infected mothers are at a high risk of acquiring the infection. The World Health Organization (WHO) recommends early diagnosis of HIV-exposed infants (HEIs) through deoxyribonucleic acid polymerase chain reaction (DNA PCR) and rapid HIV testing. Early detection of paediatric HIV is critical for access to antiretroviral therapy treatment (ART) and child survival. There's, however, limited evidence of the adherence to early infant diagnosis (EID) of HIV testing protocol among HEIs in fishing communities in Uganda. This study assessed adherence to EID of HIV testing protocol among HIV-exposed infants in a hard-to-reach fishing community in Uganda.

Methods

We conducted a cross-sectional study employing quantitative data collection methods among HEIs in selected healthcare facilities in Buvuma islands, Buvuma district. We obtained secondary data from mother-infant pair files enrolled on the EID program using a data extraction tool. Data were analysed using STATA Version 14. Modified poisson regression analysis was used to determine the factors associated with non-adherence to the 1st DNA PCR test among HIV-exposed infants enrolled into care.

Results

None of the HIV-exposed infants had done all the EID tests prescribed by the HIV testing protocol within the recommended time frame for the period of January 2014-December 2016. Adherence to the 1st and 2nd DNA PCR, and rapid HIV tests was 39.5%, 6.1% and 81.0% respectively. Being under the care of single mothers (PR=1.11, 95% CI: 1.01-1.23, p=0.023) and cessation of breast feeding (PR=0.90, 95% CI: 0.83-0.98, p=0.025) were significantly associated with non-adherence to the 1st DNA PCR.

Conclusion

None of the HIV-exposed infants adhered to all the EID tests of HIV testing protocol. Adherence to the 1st DNA PCR was positively associated with being a single mother and exclusive breast feeding. Therefore, single mothers and those who stop breastfeeding should be supported to ensure timely EID.

Keywords: Diagnosis; Infant; Hard-to-reach; HIV; Uganda

Background

HIV/AIDS is a leading cause of infant mortality in resource-limited settings worldwide [1]. Despite significant scale-up of programs to prevent mother-to-child transmission (MTCT) of HIV, over 90% of new infections among infants and young children still occur during pregnancy, child birth, or through breastfeeding [2]. More than 150,000 children were newly infected with HIV while 100,000 died from AIDS-related causes in 2020 [1, 3]. The majority of these live-in resource-limited settings in sub-Saharan Africa, where up to 30% of untreated HIV-infected children die before their first birthday, and more than 50% die before they reach 2 years of age [4]. According to the Uganda Population-based HIV Impact Assessment (UPHIA) of 2017, approximately 96,000 children in Uganda were living with HIV and more than half (54.3%) were not on ART [2]. Survival of HIV-positive infants depends on early diagnosis and treatment [5]. Unlike in adults, disease progression is rapid in HIV-positive infants [5, 6].

Owing to the risk of mortality before the age of 2 years among HIV-infected infants, the World Health Organization (WHO) recommends that national programmes should establish the capacity to provide early virological testing of HIV-exposed infants for HIV at six weeks or as soon as possible thereafter to guide clinical decision-making at the earliest possible stage. All infants with unknown or uncertain HIV exposure who are brought for healthcare at or around birth or at the first postnatal visit should get a 1st polymerase chain reaction (PCR) test within 6–8 weeks or the earliest opportunity thereafter followed by a 2nd PCR 6 weeks after cessation of breastfeeding. In addition, the Ugandan Ministry of Health (MoH) guidelines recommend a Dry Blood Spot (DBS) for confirmatory DNA PCR for all infants who test positive on the day they start ART; a DNA PCR test for all HEIs who develop signs and symptoms suggestive of HIV during follow-up, irrespective of breastfeeding status as well as a rapid HIV test at 18-24 months for all infants who test negative at 1st or 2nd PCR [7, 8].

Early infant diagnosis provides an opportunity to offer optimal and timely treatment of HIV-infected children and informs decision-making on infant feeding which improves treatment outcomes [9, 10]. Whereas EID is important in mitigating MTCT, its implementation has been challenging in resource-limited settings. For instance, more tha Delete more than [illegible]f the infants living with HIV worldwide were left undiagnosed in 2020 [3, 11]. Similarly, the HIV status was unknown for nearly two-thirds (60.6%) of the children aged 0-4 years living with HIV in Uganda

in 2017.[2] This falls way below the 95-95-95 targets of diagnosing 95% of all HIV-positive persons, enrolment of 95% of all diagnosed HIV-positive persons on ART and achieving 95% viral suppression for those on ART by 2030 [12, 13].

Current evidence shows that fishing communities in Uganda have higher HIV incidence rates and prevalence compared to the general population [14]. The fact that the HIV prevalence and incidence rates are high implies that the number of HEIs is likely to be high. This therefore requires strengthening EID in the health facilities. There’s, however, a dearth of evidence of the adherence to EID of HIV testing protocol and associated factors among HIV exposed infants in fishing communities in Uganda. Therefore, this study aimed at assessing the adherence to EID of HIV testing protocol among HIV exposed infants in a hard-to-reach fishing community in Buvuma district, Uganda. We used the Andersen and Newman’s framework to examine the factors that either facilitated or impeded utilization of EID services. According to Andersen and Newman (15), there are three key elements in the model: predisposing factors (which include, demographic characteristics, social structural variables, and an individual's basic beliefs, attitudes, and knowledge pertaining to health services), enabling (resources available, whether individually or in a community), and need-for-care factors (illnesses, conditions, and health statuses requiring health services), which either facilitate or hinder the utilization of services by individuals.

Materials and methods

Study design and area

A cross- sectional study employing quantitative data collection methods was conducted in Buvuma islands, Buvuma district, Uganda. Buvuma district is made up of 52 scattered islands in the northern shores of Lake Victoria in central region of Uganda. In 2014, Buvuma islands had a population of 89,890 people, of which 48,414 were males and 41,476 were females [16]. Fishing is the major economic activity in the area. Given that the population in Buvuma is a fishing community, it is considered to be at a high risk for transmission of HIV. Available data indicates that the HIV prevalence in Buvuma islands is as high as 11.5% , and is above the national prevalence of 6% [17]. To date, there are a number of interventions aimed at understanding the HIV status of HEIs. Through efforts by the government of Uganda and implementing partners, all health facilities have been recommended to offer EID services at both static and outreach programs such as immunization and postnatal care. The district has 12 healthcare facilities, 10 public and 2 private-not-for profit (PNFP). It has one Health centre IV, four Health centre IIIs and the rest being health centre IIs [18]. Early infant diagnosis services are offered in all the health facilities.

Study population and eligibility criteria

The study population were mother-infant pairs who enrolled on the EID program between January 2014 and December 2016 in the selected healthcare facilities. This period was chosen because infants were expected to have completed the 2-year EID cascade. Mother-infant pair files with missing data on key variables (e.g. age, sex of the infant, marital status, level of education) were excluded from the sample.

Sample size determination and sampling technique

The sample size was estimated using the Leslie Kish formula [19]

$$n = [\frac{[(z^2 * p * q) + ME^2]}{[(ME^2 + z^2 * p * q)/N]]}$$

An estimated prevalence of 32% of HEIs who complete the EID testing cascade [20] was considered, the standard normal deviate at 95% confidence (1.96), and a 5% margin of error yielded a minimum sample size of 190 mother-infant pair files. Considering a missingness of files of 40% based on a study by Gloyd, Wagenaar (21).

$$n = \frac{190}{1 - 0.4}$$

The calculated sample size was 317. The distribution of mother-infant files per selected healthcare facility was based on sampling proportionate to size as is indicated in Table 1 below.

Table 1: Distribution of mother-infant files per selected healthcare facility

**Name of Health facility**	**Total number of HEIs at the Health facility (N) (2014-2016)**	**Sample size (n)**
Buvuma Health Centre IV	160	116
Bugaya Health Centre III	62	45
Busamuzi Health Centre III	60	44
Namatale Health Centre III	55	40
Lwajje Health Centre II	49	36
Lubya Health centre II	49	36
**Total**	**435**	**317**

Sampling procedure

The health facilities were categorized depending on levels of health centre. The district has one health centre IV and three health centre IIIs. Purposively, the one health centre IV and all IIIs were selected since they are mandated to offer ART services including EID. Simple random sampling without replacement was used to select two health centre II's out of the five for geographical representation of the island. For health centre II's, their names were written on pieces of paper, folded and put in separate boxes depending on the level of care. The box was shaken such that they are mixed. A piece of paper was then picked at a time without replacement. At each level, two health facilities were selected the health facility name was written down.

Study variables and measurement

The primary dependent variable was adherence to all the EID tests of HIV testing protocol. This was a binary outcome which was defined as having done (Yes) or not having done (No) all the three EID tests within the prescribed time frame i.e. doing 1st DNA PCR at 6-8 weeks, 2nd DNA PCR at 6 weeks after cessation of breast feeding and a rapid HIV testing at 18-24 months. The recommended time for caseation of breastfeeding is 1 year for infants who turn HIV negative at the second PCR and up to 2 years for those who turn HIV positive at any level of the cascade. HEIs who tested HIV positive after taking any of the tests were considered to have adhered at that stage. An infant was considered non-adherent if they did not meet the above criteria. Percentage of HEIs enrolled into care that adhered to the EID testing protocol was calculated by dividing the total number of HEIs who had actually undergone the 1st and 2nd DNA PCR tests, and rapid diagnostic test within the recommended timeframe by the total number of HEIs in care and

multiplying by 100. The secondary dependent variable of interest in this study was non-adherence to the 1st DNA PCR. This was a binary outcome which was defined as having done (Yes) or not having done (No) the 1st DNA PCR 6-8 weeks. An infant was considered non-adherent if they did not undertake the 1st DNA PCR within 6-8 weeks. The independent variables included healthcare facility factors such as availability of supplies for EID, sample transportation system, turn-around time, linkage and follow up system, and availability of tracking tools; and Maternal characteristics such as socio-demographics (age, occupation, education, wealth status, marital status, location of residence (rural vs urban), ART status, mobility, stigma, HIV disclosure status to partner, place of delivery and distance from health facility.

Data collection procedures and tools

A review of EID registers, mother-infant pair files, ART registers and DBS dispatch forms was done to determine adherence to EID testing protocol basing on the standard of MOH guidelines. A data extraction tool was used to collect information on the health facility variables as well as infant and mother characteristics.

Data management and analysis

Data were field edited for consistence and omissions. Electronic data were transferred from Microsoft Excel to Stata® version 14 (Statacorp, College Station, TX) software for statistical analyses. Exploratory data analyses were conducted to check the consistency and cleanliness of data. Data were cleaned and assembled into analytic dataset. All the hardcopies of the completed data collection tools were secured under lock and key, and would only be accessed by only the principal investigator and the supervisor. Descriptive statistics were obtained for the categorical variables and presented as frequencies and percentages. In addition, continuous variables were summarized using measures of central tendency and dispersion.

In order to determine the factors associated with non-adherence to the 1st DNA PCR among HEIs enrolled into care in Buvuma islands, Buvuma district, categorical variables were cross tabulated to identify the proportion of cases within a subgroup. Thereafter, bivariate analysis was conducted to determine the relationship between the independent and outcome variable. Unadjusted prevalence ratios, corresponding 95% confidence intervals and p-values were obtained using ‘modified’ Poisson regression. All independent variables associated with non-adherence to 1st DNA PCR test at bivariate analysis with a p-value of less than 0.20 were considered for

multivariable analysis. “Modified’ Poisson regression analysis was used to obtain adjusted prevalence ratios given that the prevalence of non-adherence to the 1st DNA PCR was common (greater than 10%) [22]. During model building, a forward stepwise strategy was used [23, 24]. This involved a stepwise addition of independent variables into multivariable model. After adjusting for the individual independent variables, a p value of less than 0.05 was considered statistically significant.

Quality control and assurance

We recruited a total of 5 research assistants. In order to ensure quality control, all the research assistants received a two-day training on the study, research ethics, use of the data extraction tool, and were supervised during data collection and entry process. Pre-visiting the study area and pretesting of the study instruments was done to ensure the appropriateness of the questions for reliable and accurate information. The data abstraction tool was pre-tested from Busi Health Centre IV located at Busi Island, Wakiso district. This was deemed appropriate since it also serves as a hard to reach fishing community on Lake Victoria with characteristics similar to those of Buvuma islands. After the pre-test, appropriate adjustments on the tool were made before actual data collection.

Ethical approval and consent to participate

This study was reviewed and approved by the Makerere University School of Public Health Higher Degrees and Research Ethics Committee (MakSPH HDREC). Permission to conduct this study was also sought from Buvuma District Local government.

Results

Flow chart for mother-infant file

A total of 435 files were screened, among these, 125 did not have information on the variables of interest (Figure 1).

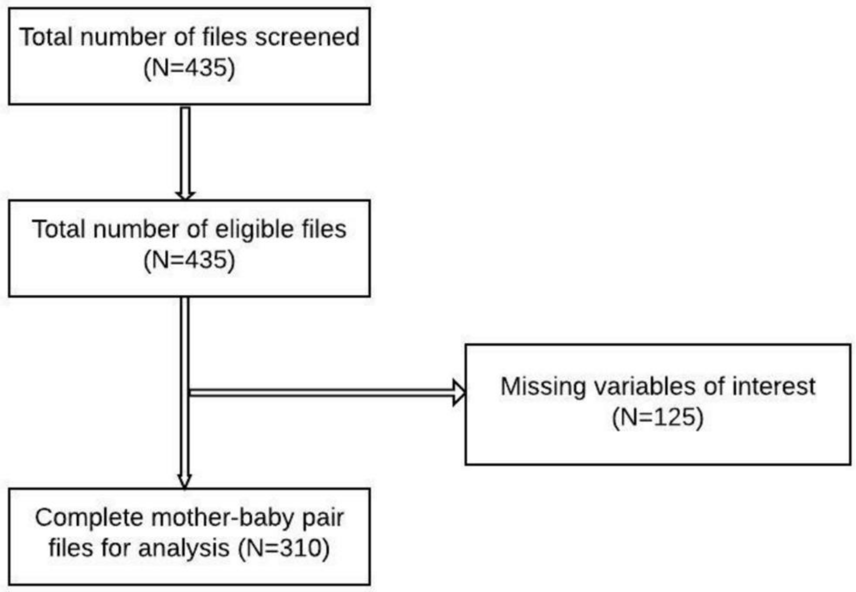

Figure 1: Flow chart on selection of study mother-infant pair files

Socio-demographic characteristics

A total of 310 mother-infant pair files were reviewed. The average age of the mothers was 28.3 (SD±5.3). About 90% (281/310) of the mothers were married and 36.8% (114/310) had been diagnosed with HIV during pregnancy. Nearly two-thirds, 61.3% (190/310) had disclosed their HIV status to their sexual partners and 67.1% (208/310) had their last delivery in a health facility. Nearly half, 47.1% (146/310) of the infants included in the sample were females (Table 2).

Table 2: Socio demographic characteristics of the study participants

Variable	Category	Frequency (N=310)	Percentage (%)
**Age category of the mothers (Mean (28.3±5.3)**	Below 20 years	16	5.2
	20+ years	293	94.8
**Marital status**	Married	281	90.6
	Single	29	9.4
**Time of HIV diagnosis for the mother**	Before pregnancy	86	27.7
	During antenatal care (ANC)	114	36.8
	During postnatal care (PNC)	81	26.1
	During immunisation outreaches	29	9.4
**Disclosed HIV status to the partner**	Yes	190	61.3
	No	17	5.5
	Missing	103	33.2
**Place of delivery for the infant**	Health facility	208	67.1
	Home	4	1.3
	Missing	98	31.6
**Distance to health facility**	≤5kilometers	144	46.5
	> 5kilometers	135	43.5
	Missing	31	10.0
**Sex of infant**	Female	146	47.1
	Male	158	51.0
	Missing	6	1.9
**Have a treatment supporter (N=241)**	Yes	230	95.4
	No	11	4.6

Sample collection and communication of EID results to mothers and caretakers

Almost all (98.1%, 304/310) the mother-infant pair files indicated that the 1st PCR sample was collected; 43.9% (132/298) indicated that the 2nd PCR sample was done while only 46.4% (137/295) indicated that the rapid diagnostic test for the infant had been done (Table 3).

Table 3: Sample collection and communication of EID results to mothers and caretakers

Variable	Category	Frequency	Percentage (%)
Was the 1st PCR sample collected? [N=310]	Yes	304	98.1
	No	6	1.9
Was the 2nd PCR sample collected? [N=298]	Yes	132	43.9
	No	166	56.1
Was the rapid diagnostic test done? [N=295]	Yes	137	46.4
	Missing	158	53.6
Result of 1st DNA PCR	Positive	12	3.9
	Negative	258	83.2
	Missing	40	12.9
Result of 2nd DNA PCR	Positive	3	1
	Negative	122	41.4
	Missing	173	58.6
Results of 1st DNA PCR communicated	Yes	241	79.3
	Missing	63	20.7
Result of 2nd DNA PCR communicated	Yes	120	40.3
	Missing	178	59.7

Average time taken to undergo EID tests and turnaround time

The average time taken by infants to undergo the 1st PCR and 2nd PCR tests was 14.5 (SD±16.8) and 61.0 (SD±29.1) respectively, while the average time taken to undergo the rapid diagnostic test was 84.1 (SD±23.4). The average time taken from testing to receiving results (turnaround time) for the 1st and 2nd PCR was 11.4 (SD±21.1) and 11.9 (SD±35.1) respectively (Table 4).

Table 4: Average time taken to conduct EID tests and turnaround time

Measures of central tendency	1st PCR testing time (weeks)	2nd PCR testing time (weeks)	Rapid diagnostic testing time (weeks)	1st PCR turnaround time (weeks)	2nd PCR turnaround time (weeks)
Mean	14.5	61.0	84.1	11.4	11.9
SD	16.8	29.1	23.4	21.1	35.1
Median	8	62	84	7	9.5
N	304	132	138	248	120

Level of adherence to EID of HIV testing protocol

Overall, none of the study participants had done all the recommended tests within the recommended time frame. About 39.5% (120/304) of the infants had done the 1st PCR test within the recommended time of 6-8 weeks, only 6.1% (8/132) had done the 2nd PCR test at the recommended time of 58 weeks while 81.0% had done the rapid diagnostic test within the recommended time of 126-168 weeks (Table 5).

Table 5: Distribution of adherence to EID of HIV testing protocol at scheduled time points

Variable	Category	Frequency	Percentage (%)
Overall adherence to all the tests (N=310)	Adherent	0	0.0
1st PCR (Between 6-8 weeks) (N=304)	Adherent	120	39.5
	Non-adherent	184	60.5
2nd PCR (at 58 weeks) (N=132)	Adherent	8	6.1
	Non-adherent	124	93.9
Rapid HIV test (18-24 months) (N=140)	Adherent	111	81.0
	Non-adherent	29	19.0

Adherence EID of HIV testing protocol based on characteristics of the study population

A higher proportion, 41.1% (113/275) of infants of married participants adhered to the 1st PCR test compared to 24.1% (7/29) their single counterparts. Close to half, 45.5% (65/143) of the female infants adhered to the 1st PCR testing protocol compared to 35.5% (55/158) of the male infants. About 41% (40.6%, 76/187) of infants of the mothers who had disclosed their HIV status to their partners and only 31.3% (5/16) of infants of mothers who had not disclosed their HIV status to their partners had adhered to the testing cascade. Nearly half, 44.7% (63/141) of the infants of clients who lived ≤5kilometers to the healthcare facility and only 36.8% (49/135) of the infants of participants who lived >5kilometers from the healthcare facility had adhered to the testing cascade. In addition, only a quarter 25.0% () of the infants of the participants who delivered from home compared to 38.1% (77/202) of those who delivered from a healthcare facility had adhered to the testing protocol (Table 6).

Table 6: Distribution of adherence to 1st DNA PCR test based on socio-demographic characteristics

**Variable**	**Category**	**Adherent N (row %)**	**Non-adherent N (row %)**	**Total**
Marital status (n=304)	Married	113 (41.1.)	162 (58.9)	275
	Single	7 (24.1)	22 (75.9)	29
Time of HIV diagnosis for the mother (n=304)	Before pregnancy	33 (39.3)	51 (60.7)	84
	During antenatal care (ANC)	42 (37.8)	69 (62.2)	111
	During postnatal care (PNC)	32 (40.0)	48 (60.0)	80
	During immunisation outreaches	13 (44.8)	16 (55.2)	29
Disclosed HIV status to partner (n=203)	Yes	76 (40.6)	111 (59.4)	187
	No	5 (31.3)	11 (68.8)	16
	Total	81 (39.1)	126 (60.9)	207
Place of delivery (n=206)	Health facility	77 (38.1)	125 (61.9)	202
	Home	1 (25.0)	3 (75.0)	4
	Total	78 (36.8)	134 (63.2)	212
Distance to the health facility (n=274)	≤ 5kilometers	63 (44.7)	78 (55.3)	141
	>5kilometers	49 (36.8)	84 (63.2)	135
	Total	112 (40.1)	167 (59.9)	279
Sex of infant (n=304)	Female	65 (45.5)	78 (54.5)	143
	Male	55 (35.5.)	100 (64.5)	158
	Total	120 (39.5)	184 (60.5)	304
Type of infant feeding (N=303)	Exclusive breast feeding	53 (34.0)	103 (66.6)	156
	No longer breast feeding	56 (47.9)	61(52.1)	117
	Complementary feeding above 6 months	11 (36.7)	19 (63.3)	30

Factors associated with non-adherence to 1st DNA PCR test

The prevalence of non-adherence to the 1st DNA PCR test was 11% higher among infants of single mothers compared to those whose mothers were married after adjusting for time of mother diagnosis for HIV, HIV disclosure status, place of delivery, distance to healthcare facility, sex of the infant and method of infant feeding (PR 1.11, 95% CI: 1.01-0.23, p=0.023). The prevalence of non-adherence to the 1st DNA PCR test was 10% lower among infants of mothers who were no longer breast feeding compared to those of mothers practicing exclusive breast feeding after adjusting for time of mother diagnosis for HIV, HIV disclosure status, place of delivery, distance to healthcare facility, sex of the infant and m (PR 0.90, 95% CI: 0.83-0.98, p=0.028). There was a borderline statistical significance between the sex of the infant and non-adherence to the 1st DNA PCR test. The prevalence of non-adherence to the 1st PCR test was 6% higher among male infants compared the females (PR 1.06, 95% CI: 0.99-1.14, p=0.088) (Table 7).

Table 7: Factors associated with non-adherence to 1st DNA PCR test among HIV exposed infants in a hard-to-reach fishing community in Uganda

Variable	Number	Adhered		Unadjusted PR (95% CI)	p-value	Adjusted PR (95% CI)	p-value
		Yes	No				
**Marital status**							
Married	275	113 (94.2)	162 (88.0)	1.0		1.0	
Single	29	7 (5.8)	22 (12.0)	1.10 (1.00-1.21)	0.038	1.11(1.01-1.23)	0.023
**Time of mother diagnosis**							
Before pregnancy	84	33 (27.5)	51 (27.7)	1.0			
During antenatal care (ANC)	111	42 (35.0)	69(37.5)	1.00 (0.92-1.09)	0.837		
During postnatal care (PNC)	80	32 (26.7)	48 (26.1)	0.99 (0.90-1.09)	0.926		
During immunisation outreaches	29	13 (10.8)	16 (8.7)	0.96 (0.84-1.10)	0.607		
**HIV disclosure status (n=207)**							
Yes	187	76 (93.8)	111 (91.0)	1.0			
No	16	5 (62.2)	11 (11.0)	1.05 (0.91-1.22)	0.429		
**Place of infant delivery (n=212)**							
Health facility	202	77(98.7)	125 (97.7)	1.0			
Home	4	1 (1.3)	3 (2.3)	1.08 (0.844-1.38)	0.536		
**Distance to facility (n=279)**							
± 5kilometers	141	63 (56.3)	78 (48.1)	1.0		1.0	
> 5kilometers	133	49 (43.7)	84 (51.9)	1.05 (0.97-1.13)	0.186	1.04 (0.86-1.12)	
**Sex of the infant (n=304)**							
Female	143	65 (54.2)	78 (43.8)	1.0		1.0	
Male	155	55 (45.8)	100 (56.2)	1.06 (0.99-1.14)	0.08	1.06 (0.99-1.14)	0.088
**Infant feeding**							
Exclusive breast feeding	156	53 (44.2)	103 (56.3)	1.0		1.0	
Cessation of breast feeding	96	51 (42.5)	45 (24.6)	0.88 (0.81-0.95)	0.003	0.90 (0.83-0.98)	0.028
Complementary feeding above 6 months	30	11 (9.2)	19 (10.4)	0.98 (0.87-1.10)	0.78	1.01 (0.90-1.15)	0.753
Never breast fed	21	5 (4.2)	16 (8.7	1.06 (0.94-1.18)	0.302	1.09 (0.96-1.24)	0.163

Discussion

This study established the levels of adherence to early infant diagnosis (EID) of HIV testing protocol among HIV exposed infants in a hard-to-reach fishing community in Buvuma district, Uganda. The study revealed that none of the study participants had done all the recommended EID tests within the endorsed timeframe. Only 39.5% of the infants had done the 1st PCR test within the recommended time of 6-8 weeks, only 6.1% had done the 2nd PCR test at the recommended time of 58 weeks and 81.0% had done the rapid diagnostic test within the recommended time of 126-168 weeks. Adherence to the 1st DNA PCR was associated with being a single mother and exclusive breast feeding of the infant.

Although none of the infants had done all the tests within the recommended timeframe, a significant proportion of mothers had completed the 1st PCR within the recommended timeframe. This may be so because the 1st PCR test is conducted at 6-8 weeks, a period when mothers are supposed to take their infants for immunisation based on the Ugandan immunisation schedule and also attend postnatal care [7, 8]. However, a lower proportion of the HEIs had completed the 2nd PCR test within the recommended time frame. This could be attributed to the fact that the 2nd PCR is conducted at 58 weeks, shortly after the completion of the immunisation schedule. The fact that mothers struggle to access health care facilities for services means that bringing their infants for the 2nd PCR test 6 weeks after immunisation is burdensome. Low proportions of HEIs who have undergone the 2nd DNA PCR test have also been reported in other parts of Uganda [25, 26].

Our study revealed that the prevalence of non-adherence to the 1st DNA PCR test was higher among infants of single mothers compared to those whose mothers were married. This could be to the fact that single mothers have limited support for transport needs as well as food and reminders to go to the healthcare facility. These have been shown to affect the mothers' motivation towards taking their infants for EID services. This is in line with the findings of Bwana et al., (2016) who reported that lack of partner support hindered adherence of infants to EID. Our findings indicate a need to sensitize mothers, especially those who are single on the significance of EID. In addition, the study found that the prevalence of non-adherence to the 1st DNA PCR test was lower among infants of mothers who were no longer breast feeding compared to those of mothers practicing exclusive breast feeding. This could be attributed to the reduced bonding between the mother and

the infant, and the fact that mother's assumption that their infants cannot contract the virus once not breast feeding.

Our study revealed that only 31.3% of infants of clients who had not disclosed their HIV status to their partners had adhered to the testing cascade compared to 40.6% of infants of clients who had disclosed their HIV status to their partners. This could be because disclosure of HIV status to partners is associated with spousal support in the form of money to facilitate transport to the healthcare facility, and reminders and accompaniment to the healthcare facility for the services. Consequently, this could influence adherence to EID. Our findings concur with those reported in Uganda which showed that HIV status disclosure by women to partners was associated with increased spousal support and increased visits to healthcare facilities [27, 28]. This indicates a need for encouraging disclosure of HIV status in order to enhance adherence to EID testing services.

Nearly half (44.7%) of the infants of clients who lived ≤5kilometers from the healthcare facility and only 36.8% of the infants of clients who lived >5kilometers from the healthcare facility had adhered to the 1st PCR test. Longer distance from the healthcare facility implies that the mother would incur higher transportation costs, which could in turn impede adherence to EID testing services. Similarly, Samson, Mpembeni (29) in a study conducted to assess the uptake of EID at six weeks after cessation of breastfeeding among HIV-exposed children in Tanzania reported that the most common reasons for non-uptake of the test mentioned by respondents were long distance from home to the healthcare facility (22.9%, 95% CI: 20.3-25.4).

Strengths and limitations

This study may have been the first to examine the adherence to EID in a hard-to-reach fishing community. Therefore, it provides useful insights into utilisation of EID testing services in such settings. The study utilises a relatively large sample size which makes the findings generalizable in a similar setting. This study was, however, affected by missing data in the patient files. Nonetheless, missingness of data had been catered for in the sample size calculation. Furthermore, findings from this study can only be applied to hard-to-reach fishing communities and may not be applicable to other hard-to- reach populations especially communities that are not mobile.

Conclusions

Our study revealed non-adherence to the EID testing protocol for all the HEIs enrolled in care from January 2014 to December 2016. A significant proportion of HEIs undertook timely rapid diagnostic test, while a few undertook timely 1st and 2nd DNA PCR tests. Slightly more than a third of the HEIs had undergone the 1st DNA PCR test, less than a tenth had undergone the 2nd DNA PCR test, and more than three quarters of HEIs had at least done the HIV rapid diagnostic test. Breast feeding and marital status were found to be associated with non-adherence to the first DNA PCR test. This study suggests the need to create awareness of the importance of adhering to the EID testing protocol among mothers, with more emphasis put on single mothers and those that cease breastfeeding.

List of abbreviations

**AIDS**	Acquired Immune Deficiency Syndrome
**ART**	Antiretroviral Therapy
**DBS**	Dry Blood Spot
**DNA**	Deoxyribonucleic Acid
**EID**	Early Infant Diagnosis
**MTCT**	Mother to Child Transmission
**HIV**	Human Immunodeficiency Virus
**HEI**	HIV Exposed Infant
**MOH**	Ministry of Health
**PCR**	Polymerase Chain Reaction
**WHO**	World Health Organization
**UPHIA**	Uganda Population-based Impact survey

Declaration

Consent for publication

Not applicable

Availability of data and materials

All relevant data are within the paper and its Supporting Information files.

Competing interests

The authors declare that they have no competing interests.

Funding

The author (s) received no specific funding for this work.

Author's contribution

NR, TS, AK and ADM conceptualised the study, participated in data collection and analysis, and participated in drafting the manuscript. AN and MN participated in the review of the data extraction form, data collection and drafting the manuscript. All authors reviewed and approved the final manuscript.

Acknowledgements

Our sincere gratitude goes to the research assistants that included Patience Oputan, Berna Nakiyagga and Jimmy Masereka for their tireless efforts during the data collection process. Special thanks go out to the healthcare providers of Buvuma District local government for providing the records from which the data were extracted.

References

1. UNAIDS. Start Free, Stay Free, AIDS Free Final report on 2020 targets 2021 [cited 2021 18th November]. Available from: https://www.unaids.org/sites/default/files/media_asset/2021_start-free-stay-free-aids-free-final-report-on-2020-targets_en.pdf.
2. Ministry of Health. Uganda population-based HIV impact assessment (UPHIA) 2016–2017. United States Department of Health and Human Services; 2019.
3. WHO. UPDATED RECOMMENDATIONS ON HIV PREVENTION, INFANT DIAGNOSIS, ANTIRETROVIRAL INITIATION AND MONITORING 2021 [cited 2021 20th November]. Available from: file:///C:/Users/user.DESKTOP-OMQ89VA/Downloads/9789240022232-eng.pdf.
4. Cook RE, Ciampa PJ, Sidat M, Blevins M, Burlison J, Davidson MA, et al. Predictors of successful early infant diagnosis of HIV in a rural district hospital in Zambézia, Mozambique. J Acquir Immune Defic Syndr. 2011;56(4):e104-e9. doi: 10.1097/QAI.0b013e318207a535. PubMed PMID: 21266912.
5. WHO. Manual on paediatric HIV care and treatment for district hospitals: addendum to the Pocket book of hospital care of children. 2011.
6. Kiyaga C, Narayan V, McConnell I, Elyanu P, Kisaakye LN, Kekitiinwa A, et al. Retention outcomes and drivers of loss among HIV-exposed and infected infants in Uganda: a retrospective cohort study. BMC Infectious Diseases. 2018;18(1):416. doi: 10.1186/s12879-018-3275-6.
7. Ministry of Health. Consolidated guidelines for prevention and treatment of HIV in Uganda. Ministry of Health; 2016.
8. WHO. Guideline on when to start antiretroviral therapy and on pre-exposure prophylaxis for HIV: World Health Organization; 2015.
9. Sam-Agudu NA, Ramadhani HO, Isah C, Erekaha S, Fan-Osuala C, Anaba U, et al. The impact of structured mentor mother programs on presentation for early infant diagnosis testing in rural North-Central Nigeria: a prospective paired cohort study. JAIDS Journal of Acquired Immune Deficiency Syndromes. 2017;75:S182-S9.
10. Dakum P, Tola M, Iboro N, Okolo CA, Anuforom O, Chime C, et al. Correlates and determinants of Early Infant Diagnosis outcomes in North-Central Nigeria. AIDS Research and Therapy. 2019;16(1):27. doi: 10.1186/s12981-019-0245-z.
11. UNAIDS. Global AIDS report -Confronting Inequalities. 2021 [cited 2021 21/09]. Available from: https://www.unaids.org/sites/default/files/media_asset/2021-global-aids-update_en.pdf.
12. Heath K, Levi J, Hill A. The Joint United Nations Programme on HIV/AIDS 95–95–95 targets: worldwide clinical and cost benefits of generic manufacture. AIDS. 2021;35(1):S197-S203.
13. UNAIDS. Understanding Fast-Track: accelerating action to end the AIDS epidemic by 2030 2015 [cited 2022 06 February]. Available from: https://www.unaids.org/sites/default/files/media_asset/201506_JC2743_Understanding_FastTrack_en.pdf.
14. Avert. New HIV infections fall by more than half in Ugandan fishing communities 2021 [cited 2021 21st November]. Available from: https://www.avert.org/news/new-hiv-infections-fall-more-half-ugandan-fishing-communities.
15. Andersen R, Newman J. Andersen and Newman framework of health services utilization. Journal of Health and Social Behavior. 1995;36(March):1-10.
16. UBOS. Uganda Bureau of Statistics (UBOS). The National Population and Housing Census 2014 – Area Specific Profile Series, Kampala, Uganda. 2017 2017 [cited 2021 29/7]. Available from: https://www.ubos.org/wp-content/uploads/publications/2014CensusProfiles/KAMPALA-KCCA.pdf.
17. UPHIA. Uganda Population-Based HIV Impact Assessment. In: Health Mo, editor. Kampla-Uganda: UPHIA; 2017.

18. MOH. COMPLETE LIST OF ALL HEALTH FACILITIES IN UGANDA 2018 [cited 2022 11th January]. Available from: https://www.health.go.ug/download-attachment/W6QpNJZbprAYZOQYMta3LeJApckIR34DNlsJQtxdaGI,.
19. Kish L. Sampling organizations and groups of unequal sizes. American sociological review. 1965:564-72.
20. UAC. UGANDA HIV/AIDS COUNTRY PROGRESS REPORT JULY 2016-JUNE 2017. Uganda Aids Commission 2017. Available from: http://www.unaids.org/sites/default/files/country/documents/UGA_2018_countryreport.pdf.
21. Gloyd S, Wagenaar BH, Woelk GB, Kalibala S. Opportunities and challenges in conducting secondary analysis of HIV programmes using data from routine health information systems and personal health information. Journal of the International AIDS Society. 2016;19:20847.
22. Zou G. A modified poisson regression approach to prospective studies with binary data. American journal of epidemiology. 2004;159(7):702-6.
23. Wilkinson L. Tests of significance in stepwise regression. Psychological bulletin. 1979;86(1):168.
24. Guidolin M, Pedio M. Forecasting commodity futures returns with stepwise regressions: Do commodity-specific factors help? Annals of Operations Research. 2021;299(1):1317-56.
25. Izudi J, Auma S, Alege JB. Early diagnosis of HIV among infants born to HIV-positive mothers on option-B plus in Kampala, Uganda. AIDS research and treatment. 2017;2017.
26. Mugambi ML, Deo S, Kekitiinwa A, Kiyaga C, Singer ME. Do diagnosis delays impact receipt of test results? Evidence from the HIV early infant diagnosis program in Uganda. PloS one. 2013;8(11):e78891.
27. Naigino R, Makumbi F, Mukose A, Buregyeya E, Arinaitwe J, Musinguzi J, et al. HIV status disclosure and associated outcomes among pregnant women enrolled in antiretroviral therapy in Uganda: a mixed methods study. Reproductive Health. 2017;14(1):107. doi: 10.1186/s12978-017-0367-5.
28. Izudi J, Okoboi S, Lwevola P, Kadengye D, Bajunirwe F. Effect of disclosure of HIV status on patient representation and adherence to clinic visits in eastern Uganda: A propensity-score matched analysis. Plos one. 2021;16(10):e0258745.
29. Samson S, Mpembeni RN, Njau PF, Kishimba RS. Uptake of early infant diagnosis (EID) at six weeks after cessation of breastfeeding among HIV exposed children: A cross sectional survey at six high volume health facilities in Iringa, Tanzania. Journal of Interventional Epidemiology and Public Health. 2018;1(10).

Click here to access/download
Supporting Information
Dataset_EID.xlsx

Click here to access/download
Supporting Information
Data abstraction form.pdf
