# Supplementary material for: Early infant diagnosis testing for HIV in a hard-to-reach fishing community in Uganda
Source: PLoS One. 2023 Jun 7;18(6):e0268416. doi: 10.1371/journal.pone.0268416 (PMC10246781; doi:10.1371/journal.pone.0268416)
Supplement: Attachment — Submitted filename: Response to Reviewers.docx [file pone.0268416.s005.docx]

**Title**: Adherence to the HIV early infant diagnosis testing protocol among HIV exposed infants in a hard-to-reach fishing community in Uganda

**Reviewer 1**

**Overview and general comments**: A well-written and important article describing adherence HIV testing protocols for early infant diagnosis in high-burden fishing communities in Uganda. Authors used a cross sectional study design and secondary data to determine factors associated with non-adherence to EID testing protocols. In general, the paper is well written with some areas for clarification. The language used throughout the paper seems to assume as if children were adult participants. The word adherence to EID seems inappropriate.

**Specific comments**: Below are a few specific comments.

**Abstract**: Very well written but includes a couple of sentences that should be edited for clarity.

**Comment**: Line 44. None of the HIV-exposed infants had “done” all the EID tests... Should be rewritten to read as “None of the HIV-exposed infants had “received” all the EID tests…

**Response**: Thank you. This has been rewritten. Page 2 Line 46

**Comment**: Line 51. None of the HIV-exposed infants “adhered” to all the EID tests of HIV testing protocol. Should be rewritten to read…None of the HIV-exposed infants “received” all the EID tests of HIV testing protocol.

**Response**: Thank you. This has been rewritten. Page 2 Line 46

**Comment**: Live 51-53. The sentence “Adherence to the 1st DNA PCR was positively associated with being a single mother and exclusive breast” should be rewritten to read as “Receiving the 1st DNA PCR was positively associated with being an infant born to a single mother and exclusively breast fed”

**Response**: Thank you. This has sentence has been rewritten. Page 2 Line 54-55

**Comment**: **Introduction**: Well written and gives the context under which the study was conducted. Author should consider moving part of the last paragraph to the methods section.

**Response**: Thank you. The description of the context has been moved to the methods section. Page 5 Lines 113-116

**Comment**: Methods: Describes the setting clearly but apart from fishing, no other risk or contextual factors such as sex work, transactional work, challenges accessing health care including the limited number of facilities and expense of travelling to them. Lack of electricity. Describing these will help readers understand the results.

**Response**: Contextual factors have been described. Lines 113-116. Page 5 Line 121-126

**Comment**: Sample size. Not sure of the formular is needed. The word “deviate” should be written as “deviation”.

**Response**: Sample size formular has been deleted. Page 6 Lines 137-143

**Comment**: The word adherence in methods should probably be changed especially as the paper describes infants.

**Response**: The word adherence has been dropped from the paper.

**Comment**: Data management: under data management, some items are described as very generically. Categorical and continuous variables could be named.

**Response**: Categorical and continuous variables have been named. Page 8 Lines 193-194

**Comment:** Results: The data is clearly presented and linked. First title “Flow chart for mother-infant file…” is incomplete and probably not required.

**Response**: First title has been removed. Page 10 Lines 226-227

**Comment:** Some of the description of results should be framed correctly since the study subjects were infants. For example, the line that reads, “Overall, none of the study participants had done all the recommended tests within the recommended time frame”. Could be written as none of the infants had received all recommended tests

**Response**: This has been changed throughout the paper.

**Comment:** Tables are very clear but are incompletely labeled. Each table should be labeled in such a way that it can be understood, should include the study area e.g. Buvuma and time period e.g. YYYY-YYYY. For example, table 5 is not well described. “ Table 5: Distribution of adherence to EID of HIV testing protocol at scheduled time points. Authors should rename all tables for clarity.

**Response**: Thank you. The captions have been updated for all tables.

**Comment:** The subtitle after table 5, has grammatical errors and should be rewritten.

**Response**: The caption has been edited. Page 13 Line 263-264

**Discussion**:

**Comment:** The last sentence of the first paragraph is not easy to understand.

**Response**: Thank you. This sentence has been edited. Lines 298-299.

**Comment:** Conclusion: well written.

**Response**: Thank you.

**Reviewer 2**

The manuscript is written well, the methodology is clear, and the analysis was made properly and support the manuscript conclusion.
I would suggest few comments:

**Comment**: In section materials and methods: Providing a map illustrating the localities of the study would be more informative

**Response:** A map showing the localities has been provided. The map has been drawn using the shape files of administrative boundaries in Uganda. We downloaded the shape files from Uganda Bureau of Statistics.

**Comment**: In results: Numbers under subtitle "Average time taken to undergo EID tests and turnaround time" should be followed by weeks

**Response:** The word weeks has been inserted in that subsection. Lines 233-237

**Comment**: In conclusions: Replace " marital status" by " non married females"

**Response:** Thank you. The sentence has been edited. Line 341
